# Association of Nociplastic Pain Features with Function in Aging Veterans with Chronic Low Back Pain

**DOI:** 10.21203/rs.3.rs-8376244/v1

**Published:** 2025-12-17

**Authors:** Victoria D Powell, Jinkyung Ha, Andrzej Galecki, Dan Clauw, Pooja Lagisetty, Maria Silveira, Caroline Logue, Sarah Krein

**Affiliations:** University of Michigan; University of Michigan

**Keywords:** Veterans, aging, chronic low back pain, nociplastic pain

## Abstract

**Background:**

Chronic low back pain (CLBP) affects nearly one million Veterans and is a leading cause of disability. Nociplastic pain, characterized by altered central nervous system processing, widespread pain and sleep disturbance, may negatively impact treatment response in this population, yet remains underrecognized.

**Objective:**

Determine the prevalence and association of nociplastic features with function among aging Veterans with CLBP.

**Design:**

Cross-sectional survey of Veterans aged 50–89 years with CLBP identified through VA administrative data. A stratified random sampling design oversampled women and Black/African-American veterans.

**Participants:**

Of 800 invited, 361 responded (45.1% response rate); after excluding those without CLBP, 342 participants were included (mean age 69.2 years, 19.2% women, 17.8% Black/African-American).

**Main Measures:**

Nociplastic features were assessed using the Fibromyalgia Survey Questionnaire to calculate a fibromyalgia severity score. Function was assessed via the Veterans RAND 12-Item Health Survey (VR-12) Physical (PCS) and Mental Component Summary (MCS) scores. Regression models adjusted for sociodemographics, comorbidities, opioid use, pain intensity, and surgical history.

**Key Results:**

Nociplastic characteristics were highly prevalent (fibromyalgia severity score mean 11.3, SD 5.9). Overall, 36% screened positive for fibromyalgia via American College of Rheumatology criteria, however, over half of respondents aged 50–64 years met criteria. Widespread pain was common; on average, pain was experienced in six discrete locations. Physical function was severely impaired (mean VR-12 PCS 30.2, nearly 2 SD below population norms); psychological function was moderately impaired (mean MCS 45). Higher fibromyalgia severity scores independently associated with poorer physical function (β= −0.30 per point increase, 95% CI −0.49, −0.12; *p* = 0.0014) and psychological function (β= −0.64, 95% CI −0.93, −0.35; *p* < 0.001) after adjustment. Gender, BMI, comorbidities, and back surgery were also associated with poorer function.

**Conclusions:**

Nociplastic features are highly prevalent in aging Veterans with CLBP and demonstrate a dose-response relationship with impaired functioning. Recognition may guide more effective, mechanism-based treatment approaches and reduce harm.

## Introduction

Chronic low back pain (CLBP) affects nearly one million US Veterans and is a common cause of functional impairment^1,2^. Aging Veterans, especially those above 50 years old, are considerably more affected than the general population^1^. Only about a quarter of older adults experience substantial improvement in back pain-related disability after a year of treatment^3^. One possible explanation for failure of certain treatments may be the unrecognized presence of nociplastic features of pain. “Nociplastic” is a recently described construct intended to specify the increasingly better understood mechanism whereby altered central nervous system processing causes a shift from pain as a straightforward perception of tissue or nerve injury (nociceptive or neuropathic pain mechanisms, respectively) to a complex emotional and threatening experience^4,5^. Nociplastic mechanisms likely underlie several chronic overlapping pain conditions (COPCs), or disorders of primary pain^6^. COPCs include conditions that frequently co-occur within individual patients, such as nonspecific CLBP, irritable bowel syndrome, fibromyalgia, and interstitial cystitis/bladder pain syndrome^6^. In COPCs, nociceptive input (such as inflammation and arthritis) may also be present but is insufficient to explain an individual’s pain intensity and interference.

Accumulating evidence suggests a dose-response relationship between reporting more nociplastic features with poorer pain treatment outcomes. After undergoing surgeries intended to relieve pain, more severe nociplastic pain characteristics predict more post-operative pain and opioid use but less clinical improvement^7–9^. In contrast to nociceptive pain mechanisms, both targeting peripheral pain generators (for example, epidural steroid injections) and opioid therapy in patients who endorse many nociplastic pain symptoms results in poorer analgesia^10–12^. Rather, individuals with prominent nociplastic features may benefit more from education, psychotherapy, structured physical activity, and certain centrally-acting medications such as duloxetine^5^. Unfortunately, nociplastic features in aging patients may be especially underrecognized. One longitudinal study found recognition delayed for a mean of seven years^13^. During this time, patients received potentially ineffective peripherally-directed treatments yet did not receive other treatments more supported by evidence. The failure to recognize and appropriately treat nociplastic features and COPCs in aging patients may predispose them to progressive functional decline, disability, and frailty.

However, it is unclear to what extent aging Veterans with CLBP, a highly prevalent COPC, report nociplastic features and whether nociplastic characteristics contribute to poorer physical and psychological function. We conducted a national survey of Veterans 50–89 years old with CLBP to test the hypothesis that more nociplastic features will be associated with worse physical and psychological functioning in a dose-dependent manner, even after adjustment for factors known to be associated with more severe functional impairment from pain (e.g. prior back surgeries, comorbidities, and pain intensity). We also hypothesized that nociplastic features will be more prevalent in later middle-age (50–64 years) vs older (65 + years) patients.

## Methods

### Study Population and Sample Selection

Using the Veterans Affairs Corporate Data Warehouse and the Medicare Vital Status File, we identified 450,896 living Veterans 50–89 years old with at least two VA provider outpatient encounters from October 2018 to September 2022 where an ICD-10 code for low back pain (M54.5, M54.40, 41, 42, M54.89)^14^ was assigned. Of those meeting selection criteria, 9.8% were women and 22% were Black/African-American (AA). The proportion of Veterans reporting Black/AA ancestry is 15.4%, and 7.4% of Veterans 50 years and older are women^27^. A stratified random sampling approach oversampled women and Black/AA Veterans by a factor of 2, identifying 800 individuals to receive study invitations. Appendix 1 contains the Strengthening the Reporting of Observational Studies in Epidemiology (STROBE) study flow diagram.

### Data Source

Study data was obtained via completion of a one-time survey. Participants could choose to complete it either electronically (via Qualtrics FedRAMP instance) or via paper, but were instructed to only complete the survey once. The packet included an invitational letter with opt-out instructions, a QR code linking to Qualtrics; a study information sheet; the paper survey with a prepaid return envelope; and a $10 gift card. Neither the paper survey nor the electronic version was individually linked. A reminder packet was sent four weeks later to all not opting out. The study protocol was approved by the VA Ann Arbor Healthcare System Institutional Review Board.

## Measures

### Back PainChronicity

All information was obtained by self-report. Participants endorsing current, ongoing back pain for ≥ 3 months were considered to have CLBP.

### Nociplastic Characteristics

Features consistent with a nociplastic pain mechanism were assessed using the Fibromyalgia Survey Questionnaire (FSQ), an adaptation of the American College of Rheumatology (ACR) fibromyalgia survey criteria for survey research^18^. The FSQ assesses pain widespreadedness (Widespread Pain Index [WPI]) by asking participants to specify discrete body pain location(s). The Symptom Severity score (SSS) assesses additional co-occurring symptoms such as fatigue and trouble thinking or remembering. The total Fibromyalgia Severity (FS) score is the sum of the WPI and SSS, ranging from 0 (symptoms absent) to 31 (most severe)^19^. Respondents were considered to screen positive for fibromyalgia if they met 2016 ACR diagnostic criteria of WPI ≥ 7 and SSS ≥ 5 *or* WPI 4–6 and SSS ≥ 9^19^. We used this instrument both to ascertain those with severe nociplastic features (defined as meeting ACR fibromyalgia screening criteria) and as a continuous measure allowing capture of those with clinically important nociplastic features not necessarily meeting a cutpoint (i.e., subthreshold symptoms). This methodology has been used in multiple studies of chronic pain populations not otherwise diagnosed with fibromyalgia, including CLBP, finding dose-dependent relationships with higher FSQ scores predictive of more disability, more opioid use (but poorer response to opioids), and poorer response to procedures intended to relieve pain, such as surgery or steroid injections^7–9,20,21^.

### Function

The Veterans RAND 12-Item Health Survey (VR-12) is a general measure of overall function and well-being extensively used in Veteran research and clinical applications^17^. The VR-12 Physical (PCS) and Mental Component Summary (MCS) scores assess physical and psychological function, respectively. Scores are standardized and normed to Veteran populations, with lower indicating poorer self-perception of overall function and well-being.

### Additional Measures

Sociodemographic data obtained included age, gender, ethnicity, race, education level, employment status, tobacco use, weight, and height (to calculate body mass index [BMI]). Items adapted from the Health and Retirement Study^15^ assessed the presence of comorbid conditions. An adapted questionnaire^16^ was used to assess surgical and opioid use history. Patient Reported Outcomes Measurement and Information System (PROMIS) short forms assessed pain intensity and interference. The PainDETECT questionnaire was used to ascertain likelihood of neuropathic mechanisms contributing to back pain^22^.

### Data Handling and Analysis

Paper survey data was double-coded and entered by two individuals then combined with Qualtrics data. Respondents without current CLBP were excluded from further analyses. Ambiguous paper survey responses (e.g. selection of multiple adjacent responses) were assigned one response via a random probability generator. For example, if a participant circled both “4” and “5” on a Likert scale, a random probability was generated; if ≥ 0.5, the response was coded as the higher value. Mutually exclusive responses (e.g. both “yes” and “no”) were coded as missing.

The HealthMeasures Scoring Service^23^ was used to generate standardized T-scores for PROMIS items. VR-12 data was scored using tool developers’ recommended code which produces a single imputation as long as a single item is non-missing^17^. Missing values in other variables were treated as missing at random. Missingness ranged from < 1% to 31%. Most items contained < 10% missing (Appendix 2). Descriptive analyses were performed on sample data. The PROC MI procedure in SAS version 9.4 (SAS Institute, Inc., Cary, NC) was used to generate and analyze 10 datasets with no missing values, imputed using fully conditional specification (FCS)^24–26^. All inferential analyses were performed on the imputed dataset. To characterize the association of nociplastic features with functioning, we constructed multiple linear regression models adjusting for age, gender, race, ethnicity, employment, education, history of back surgery, BMI, number of comorbidities, pain intensity, and prescription opioid use. R^2^ values were used to assess goodness-of-fit^26^. The MI estimate of each statistic of interest in the regression models was calculated by averaging across the 10 imputed datasets according to Rubin’s rules^26^.

## Results

In total, 361 surveys were returned or completed electronically (45.1% response rate). After excluding those without CLBP (n=19), the final sample contained 342 veterans who reported experiencing back pain for nearly 27 years on average. Most (86.5%) indicated back pain was a problem at least half the days in the past 6 months. The proportion of women (19.2%) and Black/AA (17.8%) respondents indicated adequate representation. Prescribed opioids were used by 14.0% for back pain specifically. Nearly one-third reported at least some prescribed opioid use in the last year, and approximately 15% used prescribed opioids long-term (≥3 months). Comorbidities were common, with hypertension and arthritis most frequently reported. Table 1 details respondent characteristics.

### Nociplastic Features:

Participants experienced pain in a mean of 6 (SD 3.8) discrete locations, though there was marked variability (range 0–18, median 5, IQR 3–8). The fibromyalgia severity score mean was 11.3 (SD 5.9) with a similarly wide distribution (range 0–28, median 11, IQR 7–15). When grouped by age, respondents aged 50–64 years had fibromyalgia severity scores that were 3.3 points higher on average than those 65 years and older. Overall, 36% reported nociplastic features severe enough to meet 2016 ACR fibromyalgia diagnostic criteria. However, 51% of the younger group met criteria compared to just 29% of the older respondents.

### Function:

On average, self-assessment of physical function was very poor, two SD below population norms (PCS T-score mean 30.2, SD 9.9). Respondents’ psychological status fared better; the MCS T-score mean of 45 (SD 13.4) was closer to the population average.

### Additional Pain Characteristics:

Average pain intensity was 5.7/10 (SD 2.3) but ranged from 0 (no pain) – 10 (worst pain imaginable). Both pain intensity and interference were severe in many cases, with mean T-scores greater than a standard deviation above population average. Slightly fewer than half reported back pain with likely or possible neuropathic features.

### Relationship of Nociplastic Features with Function:

**Figure 1:**
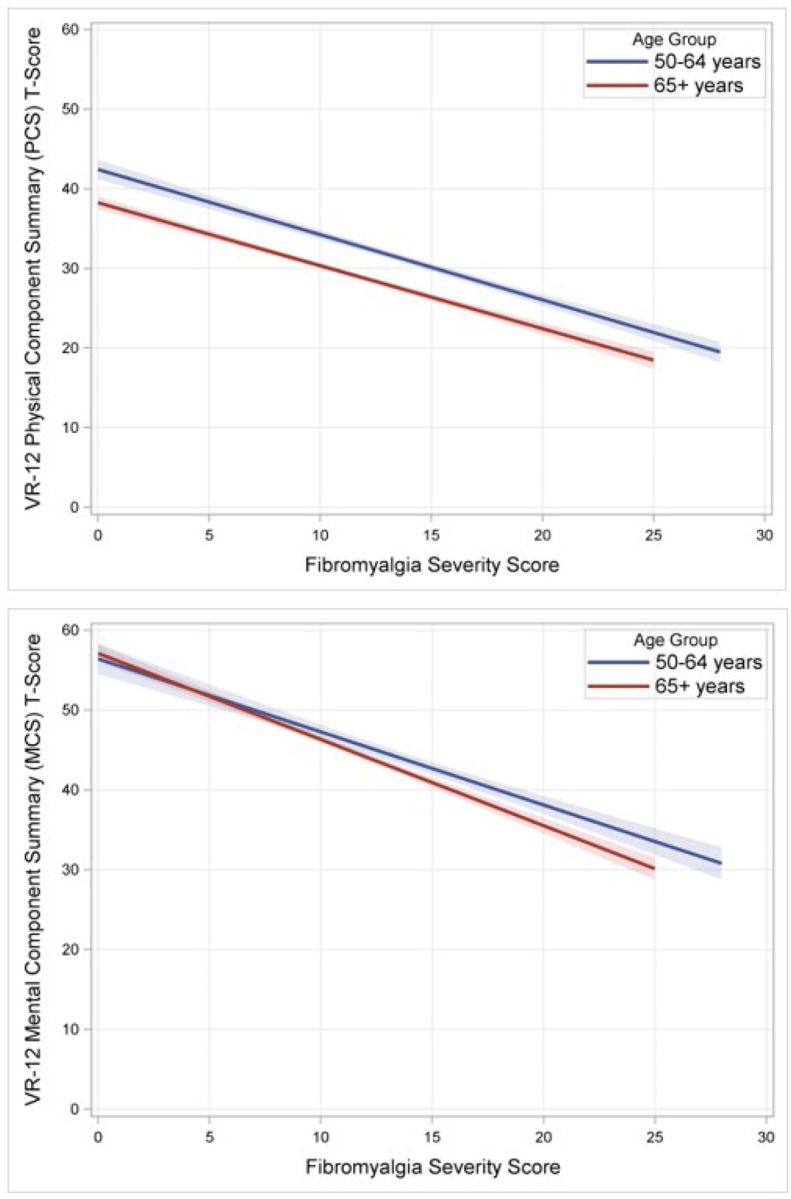
Relationship between nociplastic features and function. Shaded regions represent 95% confidence intervals. Top panel: Physical function (measured by the VR-12 PCS T-score) was poorer on average in the older (65+ years) group compared with the younger (50–64 years) group. More severe nociplastic features (as indicated by higher total fibromyalgia severity scores on the FSQ) were associated with poorer function regardless of age. Bottom panel: There was no difference in psychological function / mental health (measured by the VR-12 MCS T-score) by age group. More severe nociplastic features are also associated with poorer mental health regardless of age.

Details of regression analyses are presented in Table 2. Ten imputations produced n=3,350 complete cases available for analysis. Higher fibromyalgia severity scores (indicating more severe nociplastic features) were associated with lower significantly poorer physical function (β −0.03 (95% CI −0.49, −0.12), p=0.0014) and psychological function (β −0.64 (95% CI −0.93, −0.35), p<0.001), after adjusting for sociodemographic factors, pain intensity, surgical history, and opioid use. Altogether, more nociplastic features, higher pain intensity, higher BMI, more comorbidities, woman gender, and history of back surgery were significantly associated with poorer physical function (lower VR-12 PCS T-scores). Nociplastic features, higher pain intensity, and employment status other than employed full/part-time were significantly associated with poorer psychological function (lower VR-12 MCS T-scores). When grouped by younger age (50–64 years) vs older (≥65 years), younger respondents had significantly higher mean fibromyalgia severity scores (mean 13.6 (SD 5.9) vs 10.8 (SD 5.3), *t*(1795.1) = 13.18, *p*<.001). However, the directionality and slope of the relationship were not significantly different in the adjusted model; higher fibromyalgia severity scores were associated with poorer overall function regardless of age (Figure 1). A sensitivity analysis of complete cases from the sample produced similar results (Appendix 3).

## Discussion

In this national sample of Veterans 50 years and older with CLBP, reporting features consistent with a nociplastic mechanism of pain (e.g., widespread pain and concurrent mood and sleep disturbance) robustly associated with poorer overall function. This dose-response relationship remained significant after controlling for sociodemographic and clinical factors known to associate with poorer functioning, such as older age. More than a third reported nociplastic features severe enough to meet diagnostic criteria for fibromyalgia, the prototypical nociplastic pain disorder, though the proportion was considerably higher among younger respondents. Despite sample selection based only on a single site of pain, participants reported experiencing six distinct painful sites on average, suggesting that multiple chronic pain conditions may be present in aging Veterans with CLBP. Notably, a minority of participants reported no or very few nociplastic characteristics, including localized back pain.

Chronic nonspecific low back pain is one of multiple COPCs where nociplastic pain is considered the predominant pain mechanism. In these conditions, other pain mechanisms may be present, but they do not fully capture the pain experience either in severity or functional interference^28^. COPCs, including fibromyalgia, CLBP, and irritable bowel syndrome among others, are termed such because they commonly co-occur within individuals. The widespread pain in this national sample suggests the presence of additional COPCs in late middle-aged and older Veterans with CLBP. In this sample, nociplastic features were common, and in a significant minority, they were very severe. Similar to prior studies^7–9^, we benchmarked “severe” nociplastic features as those meeting 2016 ACR fibromyalgia screening criteria. The chronic, multisite pain experienced by patients with fibromyalgia, a condition defined by pain co-occurring alongside symptoms of fatigue, dyscognition, and unrefreshing sleep, is a prototypical example of the “nociplastic” pain category, a term coined to distinguish it from nociceptive and neuropathic pain^4,5^. When survey instruments are used to screen for fibromyalgia, prevalence ranges from 2–4% (general or non-clinical populations) to above 40% (patients presenting to a tertiary back pain clinic).^29–31^ Similar to our findings, younger age, unemployment, higher pain severity, and poorer physical function were all associated with screening positive.

Prior investigation into nociplastic characteristics among older veterans with CLBP has been extremely limited. One small study of Veterans > 65 years old noted a prevalence of approximately 25%^30^. Our finding a prevalence of about 35% may relate to our sample’s relatively younger mean age; indeed, when divided into younger (50–64 years) and older (≥ 65 years) respondents, prevalence was significantly higher in the younger group. Overall, patients with CLBP may have more prevalent and severe nociplastic features than in certain other chronic pain states. For example, only 8–9% of patients presenting for surgeries intended to relieve pain screen positive for fibromyalgia, indicating severe nociplastic features may be less prevalent^7–9^. Our findings support that persistent, nonspecific back pain is a disorder of primary pain rather than a result of peripheral pathology, such as degenerative changes seen on imaging.

Clinically, nociplastic characteristics are important to recognize because their presence predicts poorer response to certain common treatments, especially those aimed at addressing peripheral pain generators as would be appropriate for nociceptive pain. For example, in patients undergoing surgery to relieve a painful condition (e.g. knee replacement, hysterectomy), reporting more nociplastic features predicts more post-operative pain, more opioid use, and less overall improvement^7,9,32^. In clinical trials of medial branch blocks and steroid injections, treatments aimed at addressing peripheral targets, participants with symptoms consistent with a nociplastic pain mechanism experience poorer outcomes^10–12,33–35^.

Encouragingly, patients with prominent nociplastic features can benefit from targeted treatment. Grounded in a trusted clinician-patient relationship, multimodal approaches featuring both pharmacologic and behavioral treatments may be helpful^36^. In older adults, recognizing nociplastic features when seeking treatment for pain may be especially important to prevent or mitigate functional decline and frailty. Our findings suggest that older Veterans, especially those ≥ 65 years, have somewhat less severe nociplastic features on average than those in the 50–64 year age range. However, when nociplastic features are present, the association with poor function is no less prominent. Unfortunately, nociplastic features are likely underrecognized in older adults; one study^13^ found that participants ≥ 55 years old reported symptoms an average of seven years before eventually receiving an appropriate fibromyalgia diagnosis. During that time, they received non-targeted treatments that were ineffective and potentially harmful.

Though effective treatments for patients with prominent nociplastic features exist, precise recognition of pain mechanism(s) in any individual patient remains a matter of some debate. Recently, a stepped algorithmic approach to identifying nociplastic pain was recommended^4^, necessitating that pain must be chronic, widespread, and accompanied by central symptoms. Moreover, this approach requires the presence of sensory hypersensitivity, either assessed through a clinical history or evoked hypersensitivity testing. The instrument used in this study to assess nociplastic features does not explicitly ascertain hypersensitivity. However, the FSQ has been used as a continuous measure in multiple studies finding similarly that higher scores (indicating higher likelihood and severity of nociplastic pain) are associated with poorer functional outcomes^37^. The FSQ has several advantages, including brevity; it can be administered during routine clinical encounters and provide actionable information for clinical decision-making.

This study has several limitations. As a cross-sectional survey, no causal conclusions can be made regarding the association with nociplastic features and poorer function. The survey response rate (45.1%), while higher than other mailout surveys of Veterans with CLBP^16^, is less than recent surveys using mixed-mode mail and phone follow up methods^38^. There is the possibility of recall bias and unmeasured confounding. As survey responses were anonymous, it is not possible to know whether respondents completed the survey multiple times. However, we believe that this occurring frequently enough to skew results is unlikely.

## Conclusions

Our findings support that in aging Veterans with chronic low back pain, characteristics of nociplastic pain are common. Among those aged 50–64 years, nociplastic features may be more prevalent and severe than later in life. When examined along a continuum, there was a strong association with reporting more pronounced nociplastic attributes and poorer self-perception of overall physical and psychological function and well-being. Future investigations, including clinical trials of new pain interventions, should examine nociplastic features to determine the efficacy of novel treatments in this important but understudied group.

## Supplementary Material

Supplementary Files

This is a list of supplementary files associated with this preprint. Click to download.

- AppendicesJGIM121525.docx

## Figures and Tables

**Table 1.** Sample Characteristics (total n=342)*

Response Method	
Paper, n(%)	269 (78.7%)
Electronic (Qualtrics), n(%)	73 (21.3%)
Back Pain History	
Duration of low back pain in years	
Mean (SD)	26.9 (15.9)
Back pain is a problem… n(%)	
Every or nearly every day	229 (67.6%)
At least half the days	67 (19.8%)
Less than half the days	43 (12.7%)
Prior back surgery, n(%)	75 (22.1%)
Demographics and Comorbidities	
Age in years, mean (SD)	69.2 (9.1)
Age 50–64 years, n(%)	92 (30.6%)
Age ≥65 years, n(%)	209 (69.4%)
Women, n(%)	60 (19.2%)
Race, n(%)†	
American Indian/Alaska Native	9 (3.1%)
Asian	7 (2.4%)
Black/African-American	52 (17.8%)
Native Hawaiian/Pacific Islander	1 (0.3%)
White	234 (80.1%)
Hispanic/Latino ethnicity, n(%)	16 (6.8%)
BMI, mean (SD)	29.4 (5.3)
Smoking history, n(%)	
Never smoked	112 (32.8%)
Current smoker	34 (9.9%)
Former smoker	164 (48.0%)
Employment Status, n(%)	
Retired	191 (61.4%)
Employed full or part-time	59 (19.0%)
Disabled	50 (16.1%)
Other	11 (3.5%)
Education, n(%)	
High School diploma/GED or less	75 (23.9%)
Some college, Vocational/skilled trade, or Associates degree	143 (45.7%)
Bachelors, masters, or professional degree	95 (30.4%)
Comorbidities, n(%)	
High blood pressure	224 (74.2%)
Arthritis	202 (66.9%)
Diabetes	116 (38.4%)
Heart disease	97 (32.1%)
Mental health issue	85 (28.1%)
Cancer	53 (17.5%)
Chronic lung disease	41 (13.6%)
Weakened immune system	32 (10.6%)
Stroke	24 (7.9%)
Dementia	3 (1.0%)
Total number of comorbidities, mean (SD)	2.9 (1.3)
Function	
Physical Functioning‡	
T-score mean (SD)	30.2 (9.9), range 8.72 – 63.0
Psychological Functioning‡	
T-score mean (SD)	45.0 (13.4), range 12.7 – 73.0
Pain Characteristics	
Widespread Pain Index (number of painful areas)	
Mean (SD)	6.0 (3.8)
Symptom Severity Scale	
Mean (SD)	5.3 (3.0)
Fibromyalgia Severity Score, mean (SD)	
Total sample	11.3 (5.9)
50–64 years old	13.7 (6.3)
≥65 years old	10.4 (5.4)
Screen positive for fibromyalgia, n(%) ‖	
Total sample	112 (35.8%)
50–64 years old	44 (51.2%)
≥65 years old	58 (29.3%)
Average pain intensity ¶	
Mean (SD)	5.7 (2.3)
Pain intensity §	
T-score mean (SD)	63.0 (8.2)
Pain interference	
T-score mean (SD)	63.1 (7.4)
Neuropathic pain, n(%) #	
Unlikely	189 (55.9%)
Possible	74 (21.9%)
Likely	75 (22.2%)
Prescription Opioid Use	
Prescription opioid use for back pain, n(%)	
Currently Using	44 (14.0%)
Used in Past	129 (41.0%)
Never Used	142 (45.1%)
Prescription opioid use in last year (any), n(%)	88 (27.3%)
Prescription opioid use in last year for ≥3 months, n(%)	49 (15.4%)

* Percentages expressed as percent of available responses, exact n varies due to missingness

† Participants could select multiple races

‡ Physical and psychological functioning measured by VR-12 Physical Component Summary (PCS) and Mental Component Summary (MCS) T-scores respectively

§ Measured by PROMIS Short Form T-scores

‖ Via 2016 American College of Rheumatology Fibromyalgia Screening Criteria

¶ Measured with 0–10 numeric rating scale

# Measured with PainDETECT questionnaire

**Table 2.** Results of multiple linear regression*

Outcome: Physical Function (VR-12 Physical Component Summary T-score)			
Model 1 (unadjusted)†			
Variable	β (95% CI)	SE	*p*
(intercept)	38.7 (36.4, 40.9)	1.15	<0.001**
Fibromyalgia Severity Score	−0.73 (−0.90, −0.56)	0.09	<0.001**
Model 2 (adjusted)‡			
Variable	β (95% CI)	SE	*p*
(intercept)	88.6 (76.4, 100.8)	6.22	<0.001**
Fibromyalgia Severity Score	−0.30 (−0.49, −0.12)	0.096	0.0014 #
Age in years	−0.11 (−0.23, 0.01)	0.063	0.083
Women (vs. men)	−2.70 (−5.06, −0.35)	1.20	0.024 #
Race: Black/African-American	−0.22 (−2.70, 2.26)	1.26	0.861
Ethnicity: Hispanic/Latino	−0.81 (−4.58, 2.96)	1.92	0.673
BMI	−0.21 (−0.38, −0.04)	0.086	0.0141 #
Employment Status			
Other	reference	–	–
Retired	−4.04 (−8.95, 0.86)	2.50	0.106
Employed full or part time	−0.62 (−5.74, 4.51)	2.60	0.8122
Disabled	−3.27 (−8.43, 1.90)	2.63	0.2142
Number of comorbidities	−1.33 (−2.02, −0.64)	0.350	<0.001**
Pain intensity ¶	−0.51 (−0.63, −0.38)	0.065	<0.001**
Education			
High School diploma/GED or less	−1.95 (−4.34, 0.45)	1.22	0.1108
Some college, Vocational/skilled trade, or Associates degree	−1.11 (−3.14, 0.92)	1.03	0.2836
Bachelors, masters, or professional degree	reference	–	–
Prior back surgery	−2.24 (−4.30, −0.17)	1.05	0.0339 #
Prescription opioid use in last year (any)	−0.167 (−2.10, 1.76)	0.983	0.8657
Model 3 (unadjusted) §			
Variable	β (95% CI)	SE	*p*
(intercept)	56.60 (53.60, 59.61)	1.53	<0.001**
Fibromyalgia Severity Score	−0.996 (−1.23, −0.76)	0.118	<0.001**
Model 4 (adjusted) ‖			
Variable	β (95% CI)	SE	*p*
(intercept)	60.06 (40.19, 79.94)	10.12	<0.001**
Fibromyalgia Severity Score	−0.64 (−0.93, −0.35)	0.150	<0.001**
Age in years	0.13 (−0.084, 0.332)	0.106	0.2399
Women (vs. men)	−0.53 (−4.34, 3.28)	1.93	0.7841
Race: Black/African-American	0.597 (−2.94, 4.14)	1.81	0.7409
Ethnicity: Hispanic/Latino	−0.373 (−6.36, 5.62)	3.04	0.9025
BMI	0.154 (−0.11, 0.42)	0.13	0.2476
Employment Status			
Other	reference	–	–
Retired	5.78 (−1.64, 13.20)	3.78	0.1265
Employed full or part time	10.75 (3.26, 18.24)	3.82	0.0049 #
Disabled	3.89 (−3.87, 11.64)	3.95	0.3259
Number of comorbidities	−0.96 (−2.09, 0.16)	0.57	0.0921
Pain intensity ¶	−0.37 (−0.57, −0.17)	0.102	0.001**
Education			
High School diploma/GED or less	−0.52 (−4.15, 3.11)	1.85	0.7805
Some college, Vocational/skilled trade, or Associates degree	−2.25 (−5.44, 0.94)	1.62	0.1672
Bachelors, masters, or professional degree	reference	-	-
Prior back surgery	1.09 (−2.10, 4.28)	1.63	0.5033
Prescription opioid use in last year (any)	0.63 (−2.50, 3.76)	1.60	0.6915

* To conduct multiple regression, the FSC method was used to impute missing values in both continuous and CLASS variables in a dataset with an arbitrary missing pattern, producing n=3,350 complete cases after 10 imputations

† Model 1 R^2^ 0.159 – 0.183

‡ Model 2 R^2^ 0.447 – 0.471

§ Model 3 R^2^ 0.171 – 0.183

‖ Model 4 R^2^ 0.268 – 0.289

¶ Pain intensity was measured by PROMIS Pain Intensity Short Form-3 T-score.

# p-value <0.05

** p-value <0.001
